# Incoming Work-In-Progress Prediction in Semiconductor Fabrication Foundry Using Long Short-Term Memory

**DOI:** 10.1155/2019/8729367

**Published:** 2019-01-02

**Authors:** Tze Chiang Tin, Kang Leng Chiew, Siew Chee Phang, San Nah Sze, Pei San Tan

**Affiliations:** ^1^Faculty of Computer Science and Information Technology, Universiti Malaysia Sarawak, 94300 Kota Samarahan, Sarawak, Malaysia; ^2^X-FAB Sarawak Sdn. Bhd., 1 Silicon Drive, Sama Jaya Free Industrial Zone, 93350 Kuching, Sarawak, Malaysia

## Abstract

Preventive maintenance activities require a tool to be offline for long hour in order to perform the prescribed maintenance activities. Although preventive maintenance is crucial to ensure operational reliability and efficiency of the tool, long hour of preventive maintenance activities increases the cycle time of the semiconductor fabrication foundry (Fab). Therefore, this activity is usually performed when the incoming Work-in-Progress to the equipment is forecasted to be low. The current statistical forecasting approach has low accuracy because it lacks the ability to capture the time-dependent behavior of the Work-in-Progress. In this paper, we present a forecasting model that utilizes machine learning method to forecast the incoming Work-In-Progress. Specifically, our proposed model uses LSTM to forecast multistep ahead incoming Work-in-Progress prediction to an equipment group. The proposed model's prediction results were compared with the results of the current statistical forecasting method of the Fab. The experimental results demonstrated that the proposed model performed better than the statistical forecasting method in both hit rate and Pearson's correlation coefficient, *r*.

## 1. Introduction

In semiconductor manufacturing, preventive maintenance (PM) is an activity that takes the entire tool offline to carry out prescribed maintenance activity in order to maintain or increase the operational efficiency and reliability of the tool and minimizes unanticipated failures due to faulty parts [1]. However, PM downtime can be costly because it takes significantly long hours. If there are insufficient back-up tools to process the incoming Work-in-Progress (IWIP) when the tool is taken offline for PM activities, a WIP bottleneck situation will be created which affects the linearity of the WIP distribution in the line.

Reducing cycle time is one of the main goals to ensure on-time-delivery to the customers, while ensuring that the wafers have good yields. Thus, it is necessary to do proper PM planning to minimize cycle time impact while ensuring the tool is operational reliable. To achieve this goal, PM should be done when the tool group has low IWIP. However, the IWIP to a tool group has high variations as it is influenced by the conditions of the tools supplying the WIP to it, and various lots dispatching decision that changes dynamically every day.

In this paper, we present a multistep univariate IWIP prediction model to forecast the IWIP to a particular tool group in a semiconductor fabrication foundry (Fab) for the next seven days. We predict seven days ahead in this study as a requirement from the Fab. The problem domain is based on X-Fab Sarawak Sdn. Bhd., which has been abbreviated as the Fab. Long Short-Term Memory (LSTM) recurrent neural network is used in the prediction model to learn the historical incoming WIP pattern of the tool group to predict the future incoming WIP pattern of that tool group. LSTM has been used in various research areas such as traffic flow prediction [2], log-driven information technology system failure prediction to discover long-range structure in historical data [3], gesture recognition [4], voice conversion [5], and aircraft engines excess vibration events predictions [6]. To the best of our knowledge, LSTM has not yet been applied in Fab to predict IWIP. Hence, the application of LSTM in our work to perform IWIP prediction is novel. The contributions of the proposed model are summarized as follows:

- A machine learning-based approach to predict the incoming WIP for a tool group of interest in the Fab. Specifically, LSTM recurrent neural network is used as the machine learning algorithm to predict the incoming WIP.
- A simplified prediction model that is capable of modeling the dynamic environment of the Fab and delivers higher prediction accuracy than the Fab's baseline method.

The remainder of the paper is organized as follows.  Section 2 introduces the related works on-time series forecasting and their limitations.  Section 3 presents the proposed framework of this research.  Section 4 describes the experimental setup, presents the results, and discusses the major findings of this research.  Section 5 highlights the contribution of the proposed framework and concludes this paper.

## 2. Literature Review

### 2.1. Research Background

In the research domain of forecasting in semiconductor manufacturing, majority of the research works focus on forecasting the cycle time of the Fab. For instance, Wang et al. [7] proposed an automatic factor selection to improve the prediction accuracy of cycle time (CT) forecasting of wafer lots. The authors presented that the ANN input factors for the CT forecasting model in the past research works are either selected manually, or empirically, which seems arbitrary and unreliable to select input factors since it depends on artificial experience. Another approach to input selection is accomplished by selecting factors that represent condition of the Fab as a whole. The examples of these factors are the average queuing time of each wafer in the Fab, the average number of process steps completed per day, and the total wafers currently in the production line. According to the author, such approach is too complex to represent the wafer flow of the Fab since it is influence by the interactions among the equipment properties, wafer properties, product mix, and production control policies. Hence, in the authors' work, input factor selection are accomplished through analyzing the collected data without artificial experience to improve the accuracy, scalability, and comprehensibility of the CT forecasting models.

In another research, Wang et al. [8] attempted short-term cycle time forecasting in reentrant manufacturing systems. According to the authors, most previous studies focus on estimating the whole CT in long-term time scales, which predicted the output time at the at the moment the wafers enter the production stage. However, considering the long production cycle (60,000–90,000 minutes) and dynamic production environment, such long-term prediction hardly meets the needs of decision making in production control. Based on the long-term forecasting comparison, the authors justified that CT forecasting in short time scales can provide more timely advice on production control, such as rebalancing work in process, changing dispatching rules, and job prioritizations.

Scholl et al. [9] presented their work on an implementation of a simulation based short-term lot arrival forecast in a mature 200 mm semiconductor Fab, conducted in Infineon Technologies, Dresden. The authors' work forecasts the lot arrival to the defect density measurement (DDM) work centre in Infineon Technologies. The problem domain of the authors' research is similar to this research where the intention of the lot arrival forecast is to avoid PM activities when the WIP is expected to be high. However, the proposed model of Scholl et al. is very specific to Infineon Technologies and is impractical to apply in other Fab. This is mainly due to the fact that the core of the simulation engine used in the research work to perform the arrival forecast proprietary simulation engine. In addition, operating methods and products modelled are specific to Infineon Technologies, which differs from other Fab. It is also important to note that Infineon Technologies is a Fab with fully-automated wafer transportation system using robotic systems as oppose to human operators in other Fabs. The lot arrival time of a fully-automated wafer delivery system between each operation steps is highly consistent compared with human-operated delivery system. These factors made the comparison difficult across other Fabs that do not have the same system and facilities.

Another similar research work was done by Mosinski et al. [10]. In this research, the authors focused on the daily delivery predictions and a bottleneck early warning system for several machine groups in the Fab of their project partner. The forecast horizon is up to 14 days. According to the authors, the prediction is based completely on statistics extracted from historical lot data traces. The research uses the Alternative Forecast Method (AFM) which uses just one exclusive data source to extract the detailed historical lot movement information.

The forecast elements of the authors' research consists of three main stages which are data collection, statistics generation, and forecast calculation. The data collection stage collects all lots that are currently Work-In-Progress (WIP). The statistics generation step aims to calculate the cycle time from the start operation step A to target operation step B. Each lot's delivery time is estimated at the forecast stage. The forecast calculation is based on a statistical evaluation of the duration between A and B from historical data. Since the time interval differs for different products, lot grouping rules based on product characteristics need to be applied. In order to improve the forecast accuracy, another lot classification step is required to group the lots based on specific predefined lot attributes such as lot priority or the lot's tardiness.

In the statistical generation step, the weightage used in the authors' model are Fab specific and hence should be defined upfront if the model is being used in a different fab. In addition, various manual data sanitization steps are required in order to remove outliers in the data. In addition, the regeneration of the cycle time statistics is resource intensive especially with large number of lots involvement. Such limitation makes this model impractical for most Fab with large number of lots. Proper lot classification is necessary to ensure that the cycle time statistics generated are relevant. In addition, special software for lot scheduling is also required to generate the relevant data.

**Figure 1:**
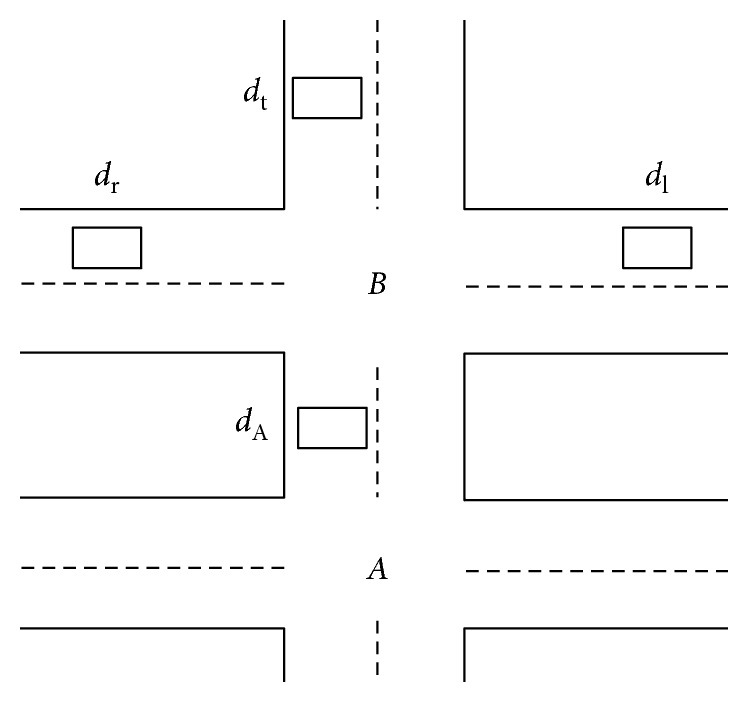
Geometric layout of traffic flow prediction scenario by Larry [6].

**Figure 2:**
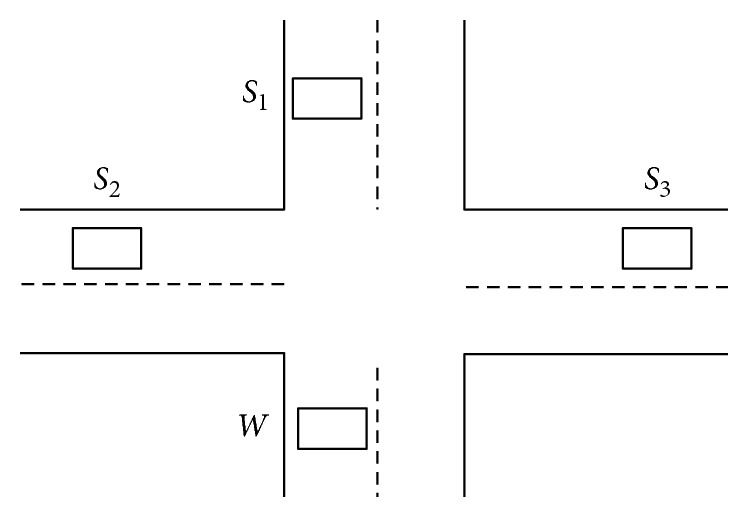
Typical scenario of WIP arrival to an equipment group in a fab.

Due to the lack of similar research works done in the domain of semiconductor fabrication, a cross-reference to similar research problem in a different domain is necessary. From the literature reviews, vehicle traffic arrival forecasting exhibits the closest similarity to the forecast of WIP arrival in the fab. Consider the comparison of the following two models presented in Figures 1 and 2.  Figure 1 presents the scenario geometry of a typical traffic arrival modelled by Larry [11], while Figure 2 presents a typical WIP arrival scenario to an equipment group in a fab.

**Figure 3:**
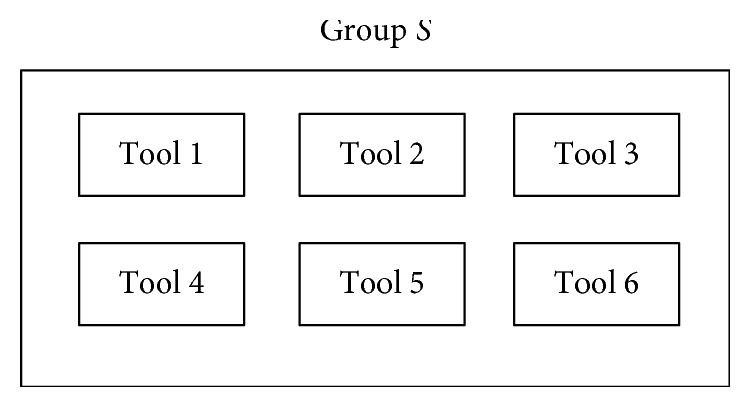
A logical grouping of tools into tool group.

In Figure 1, *d*_r_, *d*_t_, *d*_l_, and *d*_A_ each represents a traffic detector deployed at their designated location, while A and B each denotes the two intersections of the road. It is desired to predict the traffic flow approaching intersection A at detector *d*_A_ where the actual traffic flow can be measured, so that the quality of the prediction can be assessed in real-time. In Figure 2, *S*_1_, *S*_2_, and *S*_3_ each denotes the tool group that supplies lots to the equipment group W. In Fab environment, the set of tools that perform the same wafer fabrication process are commonly grouped logically and termed as tool group.  Figure 3 depicts an example with six tools in Group S.

*S*_1_, *S*_2_, and *S*_3_ are analogous to the three roads interconnected at intersection B that supplies traffic to intersection A in Larry's model [11]. The lots that are supplied by *S*_1_, *S*_2_, and *S*_3_ becomes the WIP for W. The traffic in Head's model is therefore analogous to the WIP of W in this work. Similar to the work of Larry [11], this research work would like to predict the total WIP that would arrive at W for a period of time in the future.

According to Larry [11], in general, traffic flow is a time-space phenomenon. Many of the subsequent traffic flow prediction works have also modelled the traffic flow prediction as a form of time series problem. A list of nonexhaustive related works include Tian and Pan [2], Williams and Hoel [12], VanderVoort et al. [13], Xie et al. [14], Huang et al. [15], Abadi et al. [16], Fu et al. [17], and Shao and Soong [18]. The models used by those research works can be categorized into two categories. The first category uses statistical models, while the second category uses machine learning models.

### 2.2. Prediction Models

The prediction models can commonly be divided into 2 categories, parametric models and nonparametric models. Parametric models refer to models with fixed structure based on some assumptions and the model parameters can be computed with empirical data [17]. Autoregressive Integrated Moving Average (ARIMA) is one of the most popular parametric models in time series prediction. It was first proposed to predict short-term freeway traffic in 1970s [19]. Subsequently, variants of ARIMA for time series prediction were proposed, such as Kohonen-ARIMA (KARIMA) [13] and seasonal ARIMA [12]. According to [1], these models are based on the assumption of stationary variance and mean of the time series. Kalman filter is another parametric approach to solving short-term time series traffic flow prediction [20–23]. In the most recent research, Abadi et al. [16] applied autoregressive model (AR) to predict traffic flow up to 30 minutes ahead. The authors' work uses complete traffic data, such as the historical traffic data collected from traffic links with traffic sensors, to predict the short-term traffic flow. As a case study, the authors predicted the flow of a downtown traffic in San Francisco, USA, and employed Monte Carlo simulations to evaluate their methodology. The authors reported an average prediction error varying from two percent for five minutes prediction windows to twelve percent for 30 minutes prediction windows with the presence of unpredictable events.

Nonparametric models refer to models with no fixed structure and parameters [1]. It is also known as data-driven model. Nonparametric models have gained much attention in solving time series problem because of the models' ability to address stochastic and nonlinear nature of time series problem compared to parametric models [17]. Artificial neural network (ANN), support vector machine (SVM), and deep-learning neural networks are examples of nonparametric models. The discovery of deep-learning neural network [24] and its reported success [25] have drawn many researchers' attention to apply deep-learning neural network to solve various research problems. Dimensionality reduction of data [26], natural language processing [27], number recognition [28], object detection [29], and organ detection [30] are examples of published research works that have demonstrated the successful use of deep-learning neural network.

LSTM, a variant of deep-learning neural network, has recently gained popularity in traffic flow prediction. In [31], Duan et al. have constructed 66 series of LSTM neural network for the 66 travel links in their data set, and validated that 1-step ahead travel prediction error is relatively small. In [32], Zhao et al. evaluated the effectiveness of LSTM in machine health monitoring systems by sampling data over 100 thousands time steps sensory signal data and evaluating them over linear regression (LR), support vector regression (SVR), multilayer perceptron neural network (MLP), recurrent neural network (RNN), Basic LSTM, and Deep LSTM. The results showed that deep LSTM performs the best among the evaluated methods. According to the authors, LSTM do not require any expert knowledge and feature engineering, as required by the LR, SVR, and MLP, which may not be accessible in practice. In addition, with the introduction of forget gates, LSTM is able to capture long-term dependencies; thus, it is able to capture and discover meaningful features in the signal data.

In [2, 32, 33], the authors reported that LSTM and Stacked AutoEncoders (SAE) have better performance in traffic flow predictions than the traditional prediction models. According to [32] also, LSTM reported to have better performance than SAE. In addition, the comparison performed in [14] for LSTM, gated recurrent units (GRU) neural network, and auto regressive integrated moving average (ARIMA) in traffic prediction had demonstrated that the LSTM and GRU performed better than the ARIMA model.

In [17] also, Tian and Pan demonstrated the use of LSTM to achieve higher traffic prediction accuracy in short-term traffic prediction as compared to [31, 34]. The authors found that the mentioned models require the length of the input historical data to be predefined and static. In addition, the model cannot automatically determine the optimal time lags. With the use of LSTM, the author demonstrated that LSTM can capture the nonlinearity and randomness of the traffic flow more effectively. Furthermore, the use of LSTM also can overcome the issue of back-propagated error decay through memory blocks, thereby increasing the accuracy of the prediction.

## 3. Methodology

The daily IWIP to a tool group is a form of time series data. This is because it is a sequence of values that observed sequentially in time. The IWIP forecast to a tool group is similar to traffic arrival forecast, where the objective is to ensure that there is enough capacity for the traffic to flow through with minimum obstruction for a given time-frame in the future in order not to create any bottlenecks in the traffic flow. The amount of WIP arriving to a tool group is analogical to the number of vehicles arriving to a road junction or a group of interlinks of interest. This research also requires multistep ahead forecasting approach as the research problem requires forecasting the IWIP multiple days ahead from the last observation in order to plan for PM activities.

### 3.1. Statistical Incoming WIP Forecasting Method

The existing solution in the Fab uses a basic statistical forecasting approach to forecast the WIP arrival for all tool groups for the next 7 days. The forecast is run once a week at the beginning of each week. The calculation steps of statistical forecasting approach are summarized in Table 1.

The existing forecasting method only caters to products with the number of wafers ordered dominating the total WIP in the production line. This is because the calculation requires the number of operation steps and their respective TAT to calculate the forecasted arrival steps. The forecasted results are therefore not accurate because the number of wafers considered in the calculation differs from the actual number of wafers in the production line. In addition, the method cannot predict the IWIP to a particular tool group more accurately because it does not include any algorithms to capture the time-dependency relation between the data. This limits the ability of the Fab managers. Fab manager refers to personnel who is assigned with the management responsibility to oversee various aspect of the Fab to ensure that the fab's production line performs smoothly to create a better PM activities schedule that could minimize negative impact to the production line. Therefore, it is important to create a forecasting model with better accuracy to assist Fab managers to carry out more effective PM activities planning that can minimize the impact on CT.

### 3.2. Long Short-Term Memory

The long short-term memory (LSTM) was developed in 1997 by Hochereiter and Schmidhuber [35] to address the exploding and vanishing gradient phenomena in RNN. The presence of these two phenomena had caused RNN to suffer in the inability to record information for longer period of time [18]. In other words, RNN is not able to capture long-term dependencies [32]. The solution to this problem is the introduction of forget gate into the neural network to avoid long-term dependencies. The forget gate is used during the training phase to decide when information from previous cell state should be forgotten. In general, LSTM has three gates, namely, the input gate, the forget gate, and the output gate. The key feature of LSTM is its gated memory cell and each cell has the above mentioned three gates. These gates are used to control the flow of information through each cell.

Let time be denoted as *t*. At time *t*, the input to a LSTM cell is *x*_*t*_ and its previous output is *h*_*t*−1_. The cell input state is $\tilde{C_{t}}$, the cell output state is *C*_*t*_, and its previous state is *C*_*t*−1_. Input gate at time *t* is *i*_*t*_, forget gate is *f*_*t*_, and output gate is *o*_*t*_. According to the structure of the LSTM cell, *C*_*t*_ and *h*_*t*_ will be transmitted to the next cell in the network. To calculate *C*_*t*_ and *h*_*t*_, we first define the following 4 equations.

Input gate:

(1) $$\begin{array}{c} i_{t}=\sigma \left(W_{i}x_{t}+W_{i}h_{t-1}+b_{i}\right). \end{array}$$

Forget gate:

(2) $$\begin{array}{c} f_{t}=\sigma \;\left(W_{f}x_{t}+W_{f}h_{t-1}+b_{f}\right). \end{array}$$

Output gate:

(3) $$\begin{array}{c} o_{t}=\sigma \left(W_{o}x_{t}+W_{o}h_{t-1}+b_{o}\right). \end{array}$$

Cell Input:

(4) $$\begin{array}{c} \tilde{C_{t}}=\tan h\left(W_{C}x_{t}+W_{C}h_{t-1}+b_{C}\right), \end{array}$$

where *W* = weighted matrices, *b* = bias vector, *σ* = sigmoid function, and tan*h* = hyperbolic tangent function.

Sigmoid function, *σ*(*x*), is defined as follows:

(5) $$\begin{array}{c} \sigma \left(x\right)=\frac{1}{1+\exp\left(-x\right)}. \end{array}$$

Hyperbolic tangent function, tan*h*(*x*), is defined as follows:

(6) $$\begin{array}{c} \tan h\left(x\right)=\frac{\exp\left(x\right)-\exp\left(-x\right)}{\exp\left(x\right)+\exp\left(-x\right)}. \end{array}$$

Using equations (1), (2), and (4), we calculate the cell output state using the following equation:

(7) $$\begin{array}{c} C_{t}=\left(f_{t}\;*\;C_{t-1}\right)+\left(i_{t}\;*\;\;\;\tilde{C_{t}}\right). \end{array}$$

Lastly, the hidden layer output is calculated using the following equation:

(8) $$\begin{array}{c} h_{t}=o_{t}*\tan h\left(C_{t}\right). \end{array}$$

**Figure 4:**
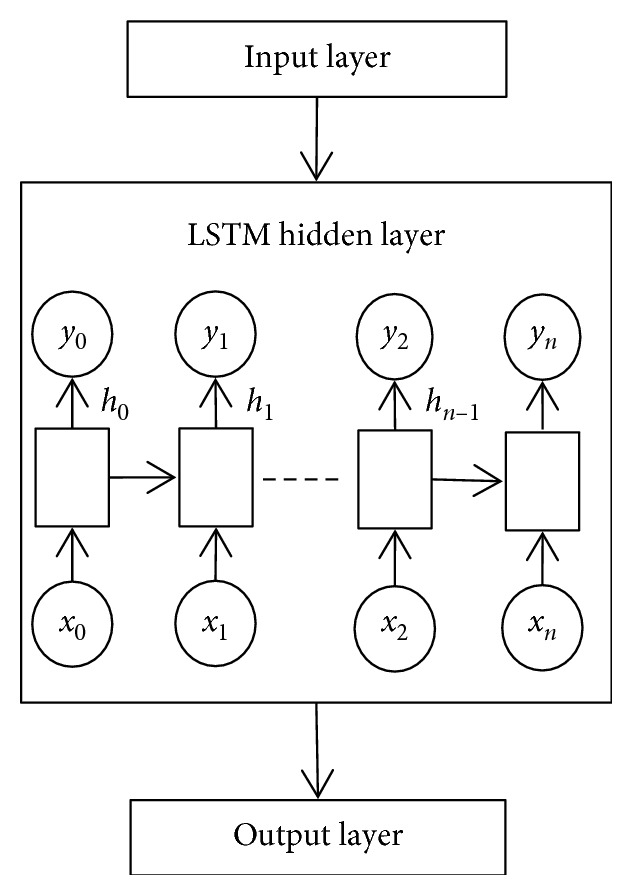
Nonstacked LSTM neural network.

**Figure 5:**
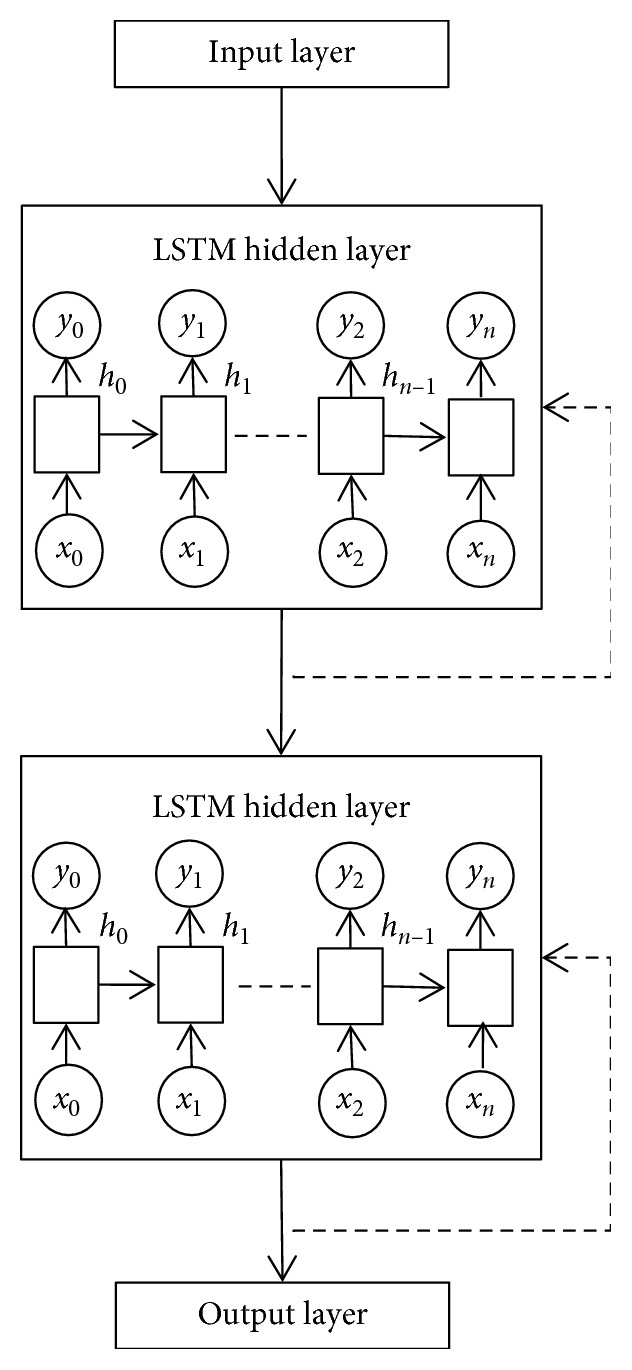
Stacked LSTM neural network.

The hidden layer of the LSTM can be stacked such that the architecture of neural network consists of more than one LSTM hidden layers.  Figure 4 shows neural network architecture with one LSTM hidden layer, while Figure 5 shows neural network with two LSTM hidden layers stacked.

With reference to Figure 5, each LSTM hidden layer is fully connected through recurrent connections (the connection is indicated by the dotted directional line). The squares in the LSTM hidden layer represents the LSTM neurons, the circles denoted with *x*_*i*_ represents the input to the LSTM neuron, while the circles denoted with *y*_*i*_ represents the output of the LSTM neuron. When the LSTM hidden layers are stacked, each LSTM neuron in the lower LSTM hidden layer is fully connected to each LSTM neuron in the LSTM hidden layer above it through feedforward connections, which is denoted by the solid directional line between the stacked LSTM hidden layers.

### 3.3. Proposed Method

**Figure 6:**
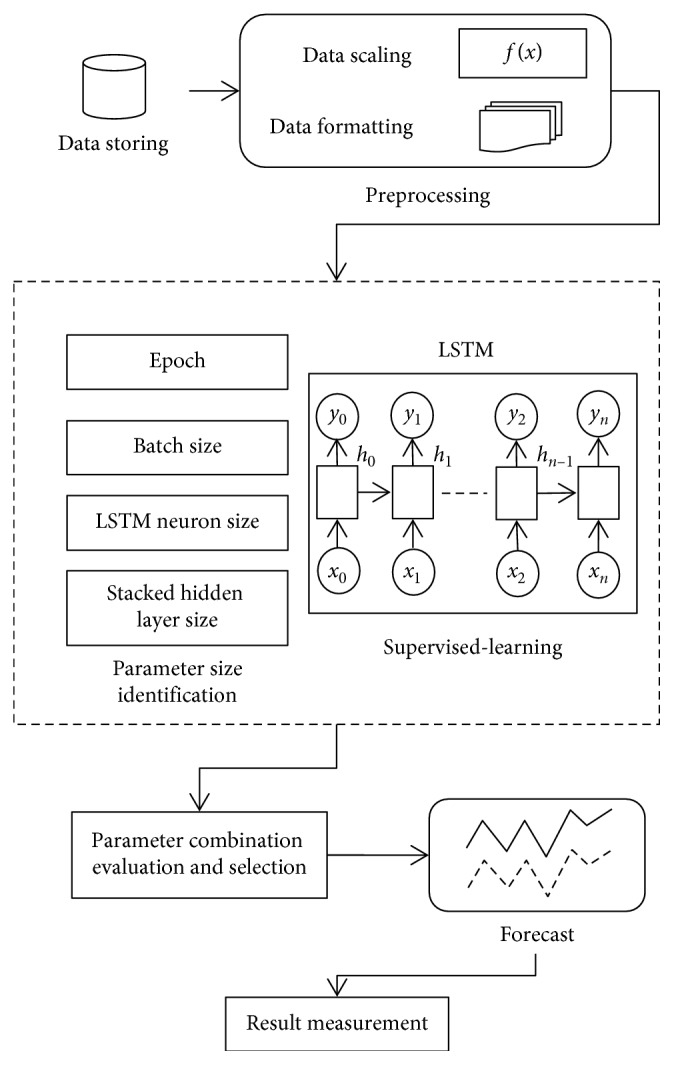
Proposed method.

Figure 6 illustrates the proposed method. The historical IWIP data are first stored in a data store to ease the management of the data. The historical data are then extracted from the data store to be preprocessed. The preprocessing stage consists of two steps, which are data scaling and data formatting.

In the data scaling step, the historical IWIP data to be used for supervised-learning is scaled according to the following equation:

(9) $$\begin{array}{c} y=\;\;\frac{x-\min}{\max-\;\;\min}, \end{array}$$

where *x* denotes each of the historical IWIP value, min denotes the smallest historical IWIP value in the historical data, and max denotes the largest historical IWIP value in the historical data.

The next step is the data formatting step. In time series domain, the term “lags” is commonly used to denote values of time steps observed prior to the prediction. Generally, the time series data are separated into training and testing set where the training set contains the lags, while the testing set contains the actual values of future time steps. Therefore, in the data formatting step, let *x*_*i*_ denotes each individual lag, the scaled historical IWIP data are formatted according to the format tabulated in Tables 2 and 3. Tables 2 and 3 depict the format of the training and testing dataset, respectively.

Following this format, column *X* consists of a series of lags, column *Y* consists of the number of time steps to be forecasted, and column *Z* consists of the number of features used in the forecast. Each row in column *X* will contain a set of seven IWIP points that corresponds to the number of IWIP points to be forecasted. These seven IWIP points are grouped into a single set of value. The number of IWIP points to be forecasted is represented in column *Y* as time steps. Column *Z* has the value of one in the training dataset, which corresponds to the single set of seven IWIP points in column *X*. By putting seven IWIP points in both the training and testing dataset, we are effectively telling LSTM that each future seven IWIP points are related to its immediate previous seven IWIP points.

As a nonparametric model, neural network model does not have a fixed structure. In a RNN with one hidden layer, the ability of the neural networks to discover important relationship in the training data during the supervised-learning is affected by the batch size used per epoch, the number of epoch, hidden layers, and hidden neuron. The combination of the sizes of these four parameters that results in stable supervised-learning and delivers the lowest forecast error is desired.

Each parameter being examined will have a list of predefined sizes to be tested. When one of the parameters is being examined, the remaining two parameters will be fixed to their current sizes in their respective list. This is to control the variation across the examinations. For each combination of the parameters, the model will be tested with that combination to measure its performance in terms of the forecasting error and the stability of its supervised-learning.

For the LSTM setup of this research, we construct a LSTM model using the LSTM cell. Let *t* denote the observation time of each IWIP and *x* denotes the IWIP, the input of the LSTM model is the observed IWIP *x* at time *t*, denoted as *x*_*t*_, and the output of the LSTM model is the predicted IWIP ${\tilde{x}}_{t+1}$. Through the LSTM equations presented, ${\tilde{x}}_{t+1}$ is therefore calculated as

(10) $$\begin{array}{c} {\tilde{x}}_{t+1}=\;\;W\cdot \;\;h_{t}+b, \end{array}$$

where *W* is the weight matrix between the output layer and the hidden layer.

The metric used to measure the forecasting error in the supervised-learning is the root-mean-squared error (RMSE). Let *P* denote the actual IWIP, $\tilde{P}$ denote the forecasted IWIP, and *n* denotes the total day forecasted, RMSE is defined as follows:

(11) $$\begin{array}{c} \text{RMSE}=\sqrt{\frac{1}{n}\overset{n}{\underset{j=1}{\sum }}{\left(P_{j}-\tilde{P_{j}}\right)}^{2}}. \end{array}$$

RMSE is a frequently used evaluation metric because it measures the difference between the values predicted by a model and the actually observed values.

For each parameter size combination to be tested, the model will be experimented multiple times with the same parameters setting. If *N* denotes the number of times the experiment was conducted, there will be *N* number of RMSE obtained to represent the performance of the model for each experiment. The reason running multiple experiments for each parameter size combination is because internally, neural network uses randomization to assign the weights and the states of its neurons. This produces different forecasting errors between experiments. Therefore, multiple experimental runs are recommended to allow for the selection of the neural network model with internal settings to produce the lowest RMSE.

**Figure 7:**
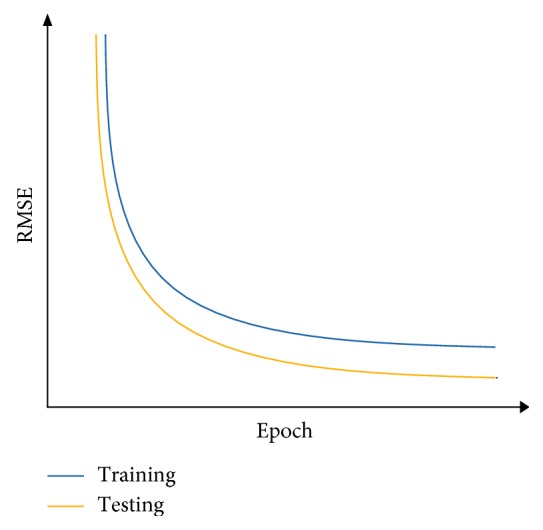
RMSE curves when the model is well learned.

**Figure 8:**
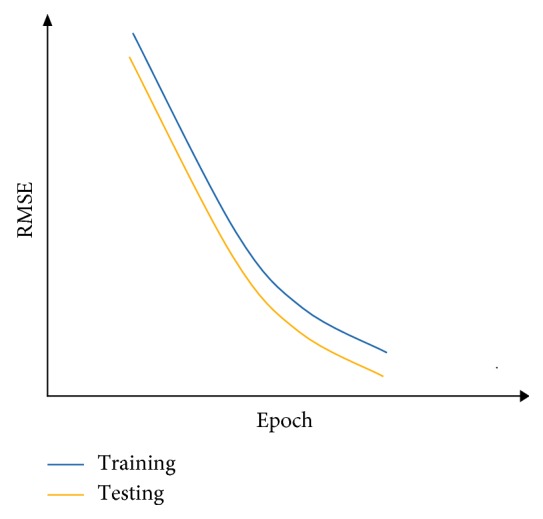
RMSE curves when then model is underlearned.

**Figure 9:**
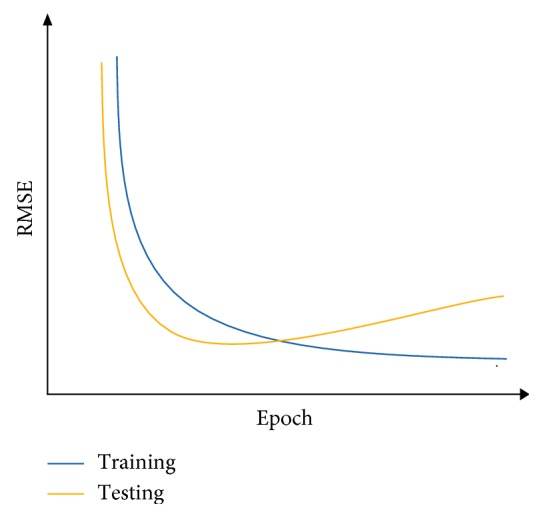
RMSE curves when the model is overlearned (overfitting).

After the supervised-learning is completed, the proposed method will proceed to parameter combination evaluation and selection. The evaluation of parameter combination and selection step is necessary because it is common to assume that a particular parameter combination that gives a low RMSE at the end of the supervised-learning directly translated to a good parameter combination that allows sufficient capability of the model to perform forecast. However, this assumption is misleading because a model that has overlearned during the supervised-learning can deliver results with very low RMSE at the end of the training. An overlearned model will perform poorly in the actual forecast. Therefore, it is necessary to also measure the stability of the supervised-learning of the model, given a particular combination of the four parameters. During each epoch in the supervised-learning, the model will be required to perform two forecasts: one uses a reserved set from the training set and other uses a reserved set from the testing set. With two forecasts performed, two RMSE will be generated. The RMSE generated by using training set is the training error, while the RMSE generated by using the reserved testing set is the testing error. To measure the stability of the supervised-learning, the RMSE for both training error and testing error of each epoch are collected and plotted in a single graph. With *y*-axis representing the RMSE and *x*-axis representing the number of epochs, Figures 7–9 show examples of the curves that exhibited from the supervised-learning. The combination of parameter sizes that allows the model to exhibit learning curve pattern similar to Figure 7 is the desired selection. Learning curve with pattern similar to Figure 7 signifies that the model was able to perform stable supervised-learning with stable reduction in the RMSE of both training and testing phases using the selected combination of parameter sizes. In other words, the model was able to discover the time-dependent relation in the given dataset such that it allows the model to minimize its prediction error for each epoch of the supervised-learning.

The combination of the four parameters' sizes that enables the model to show stable performance in the supervised-learning and lowest RMSE will be selected to forecast the IWIP.

For each of the selected parameter combination, the model is required to forecast for three consecutive weeks. The accuracies for the forecast results will be measured according to the selected measurement metrics to evaluate the forecasting capability of the model.

## 4. Experimental Results

### 4.1. Data Description and Experimental Design

The IWIP for a particular tool group is denoted as IWIP and can be calculated as

(12) $$\begin{array}{c} \text{IWIP}=\left({\text{WIP}}_{t24}-{\text{WIP}}_{t1}\right)\overset{24}{\underset{t=1}{\sum }}\left(\text{MOVE}\right), \end{array}$$

where MOVE denotes the number of wafer moved per hour, *t*1 refers to the first hour the data are collected, and *t*24 refers to the twenty-fourth hour the data are collected. In this study, the first hour is at 0830 while the 0730 on the next day is the twenty-fourth hour.

The data use for this experiment is acquired from the Fab's internal development database, with the application running hourly to collect the WIP and calculate the number of wafers moved for each tool group in the production line every 24 hours. Due to the Fab's data security and confidentiality policies, we are only allowed to access production system's data source of the company to perform data collection for a specific duration. Given the allowed duration from the Fab, we were able to collect three months data to create a data set with 90 days of historical IWIP. With each IWIP as a data point, 70 percent of the data points are used for the LSTM training phase and the remaining 30 percent for testing phase.

For the number of epochs, numerical values of 100 and 200 are selected. For batch size, numerical values of 10 and 20 are selected; for the number of hidden layers, numerical values of 3 and 4 are selected, and for the number of hidden neuron, numerical values of 384 and 512 are selected for the first hidden layer, while the numerical values of 8 and 16 are selected for the subsequent layers. It is worthwhile to mention that by using seven IWIP points per dataset as the number of previous IWIP lags to be examined, each of the numerical values for batch size denotes the number of weeks presented to the LSTM model per epoch. The neural network is initialized with uniformly distributed weights where the ranges of the weights are (−0.1, 0.1) and trained using mean-squared-error (MSE) as the loss function. Adam optimizer is used as the optimization function with default learning rate, *η*=0.001, *β*_1_=0.9, *β*_2_=0.999, *ε*=0, and *γ*=0. Each combination of the selected values is then evaluated three times to obtain the three RMSE results of each combination.

Parameter size selection is done by selecting the lowest RMSE among the three experimental runs followed by examining the graphs of the supervised-learning result of the same run that produced the lowest RMSE. The desired supervised-learning graph should resemble the pattern illustrated in Figure 7. The parameter size combinations that do not meet the required pattern will be discarded.

### 4.2. Measurement Metrics

To measure the performance of the models, two accuracy measurements are used. These two-measurement metrics are hit rate and correlation measurement.

Hit rate or probability of detection (POD) is the probability that the forecasted event matches the observed event. In the context of this research work, the observed events are either low IWIP or high IWIP. Therefore, hit rate can be used to measure the forecast capability of the proposed method to match the actual IWIP events. Let HR denotes hit rate, *n*  denotes the number correct detection, and *N* denotes the total number of observation, hit rate is expressed as

(13) $$\begin{array}{c} \text{HR}=\;\;\frac{n}{N}\;\;\times 100\%. \end{array}$$

From the requirement of the Fab, it is only necessary for the proposed method to be able to forecast any two days with highest IWIP and any two days with lowest IWIP. For these four days to be forecasted, the hit rate required by the Fab is 75 percent. In other words, at least three out of these four days must be detected.

To measure the correlation between the actual IWIP and the forecasted IWIP, this research uses the Pearson's correlation coefficient, *r*. Pearson's *r* is a measure of the linear relationship between two vectors of variables. In this research work, these two vectors of variables are the actual IWIP and the forecasted IWIP. Let *y* denotes the actual IWIP and $\tilde{y}$ denotes the forecasted IWIP, Pearson's *r* is expressed as

(14) $$\begin{array}{c} r=\frac{\mathrm{cov}\left(y,\;\;\tilde{y}\right)}{\sigma_{y}\sigma_{\tilde{y}}}, \end{array}$$

where cov is the covariance of actual IWIP and forecasted IWIP, *σ*_*y*_ is the standard deviation of the actual IWIP, and $\sigma_{\tilde{y}}$ is the standard deviation of the forecasted IWIP.

The correlation coefficient takes values in the range [−1, 1]. The value of 1 implies that a linear equation describes the relationship between the two vectors perfectly. This means that all data points of the two vectors fit perfectly on a straight line on a graph. The positive sign of the coefficient indicates positive correlation. This means that as the actual IWIP increase, and the forecasted IWIP increases as well. The negative sign of the coefficient indicates negative correlation. This means that, as the actual IWIP increase, the forecasted IWIP decreases as well. Positive correlation is therefore desirable for the forecast results.

Due to the Fab's privacy protection agreement, only the obtained Pearson's *r* will be reported, while the detail calculations of the covariance and standard deviation will be omitted. Based on the requirement of the Fab, the minimum Pearson's *r* value is 0.4.

We conduct the experiment for three consecutive weeks. This allows us to monitor the consistency of the models' prediction. At the beginning of each week, we will predict seven days ahead and measure the performance at the end of each week. The implementation of the proposed method is accomplished using Python programming language and Keras [36] neural network library.

### 4.3. Results Analysis and Discussions

Table 4 tabulates the results of the experiments. The parameter size combinations obtained in Table 4 are combinations that exhibited curve pattern similar to Figure 7.

**Figure 10:**
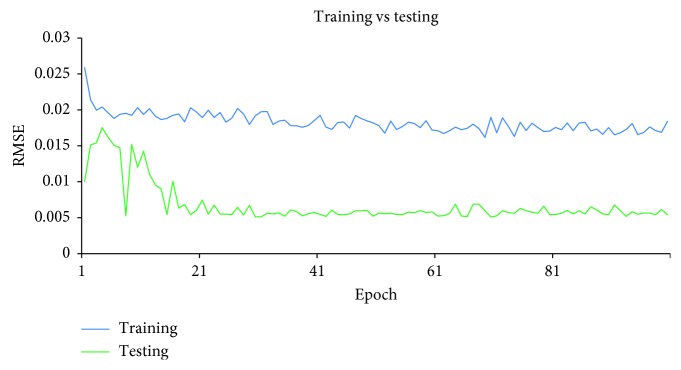
Supervised-learning result for Combination 1.

**Figure 11:**
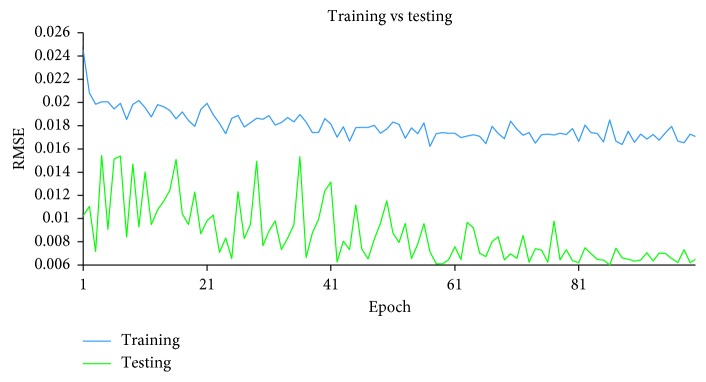
Supervised-learning result for Combination 2.

**Figure 12:**
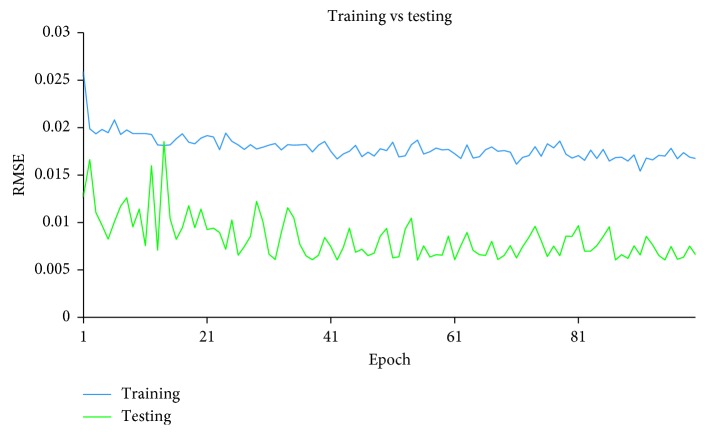
Supervised-learning result for Combination 3.

Figures 10–12 show the graphs of the supervised-learning results for the selected three combinations, respectively. From the figures, it can be seen that both lines are far apart although they move along in descending pattern. In addition, the line graph of the training is descending slowly and remained high at the end of the epoch. However, the neural network was still able to forecast the IWIP that are quite close to the actual IWIP. This is shown by the line graph of the testing's RMSE that exhibits small fluctuates. By referring to these figures alone, we are not able to identify the best parameter size combination because all three graphs exhibit similar pattern. Therefore, hit rate and linear correlation of the forecasting results of each combination can be used to identify the best parameter size combination.

The parameters from each of the selected three combinations were applied on the proposed LSTM model to perform the three consecutive weeks forecasting. The experiments were run and recorded separately for each combination. Tables 5–7 tabulate the hit rate percentage for Combinations 1, 2, and 3, respectively. Tables 8–10 tabulate the Pearson's *r* for Combinations 1, 2, and 3, respectively.  Table 11 summarizes the hit rate of Combinations 1, 2, and 3, while Table 12 summarizes the Pearson's *r* of Combinations 1, 2, and 3.

**Figure 13:**
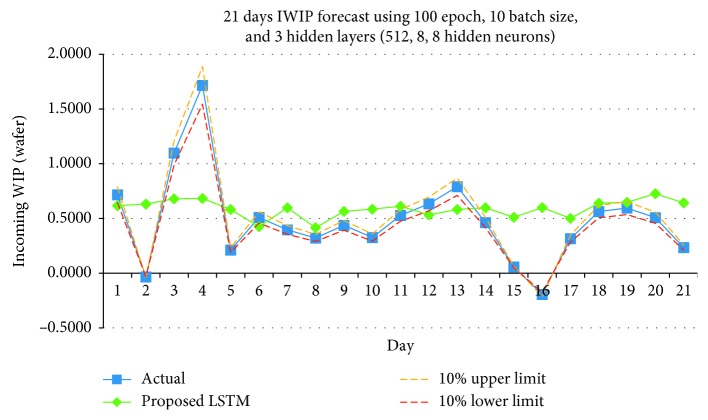
IWIP forecast using Combination 1.

**Figure 14:**
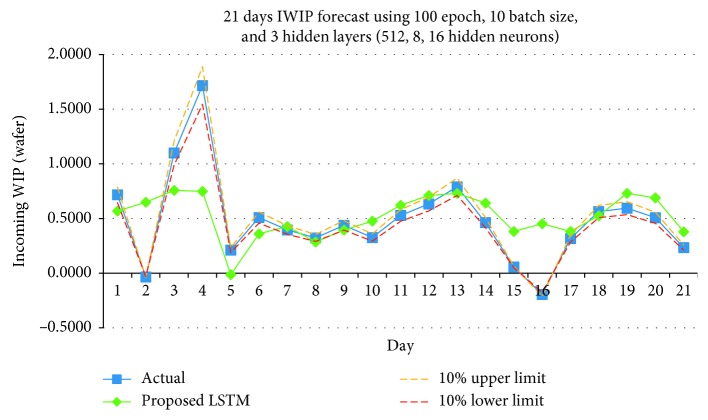
IWIP forecast using Combination 2.

**Figure 15:**
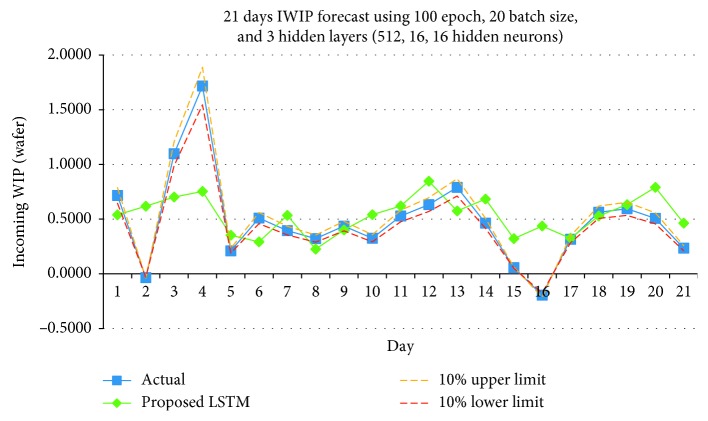
IWIP forecast using Combination 3.

Figures 13–15 shows the graph plots for the IWIP forecast for the three parameter size combinations, respectively.

From the results obtained, the model performed the best using Combination 3. In terms of hit rate, Combination 3 scored the highest compare to Combinations 1 and 2 for all three weeks. Combinations 1 and 2 scored 75 percent for week 1, but for subsequent weeks, both combinations only scored the maximum of 50 percent. In terms of Pearson's *r*, Combination 3 has the best performance in overall compare to Combinations 1 and 2, while Combination 1 has the least performance. Although on week 3, the Pearson's *r* of Combination 3 is slightly lower than Combination 2; however, it is still above the Fab's requirement.

**Figure 16:**
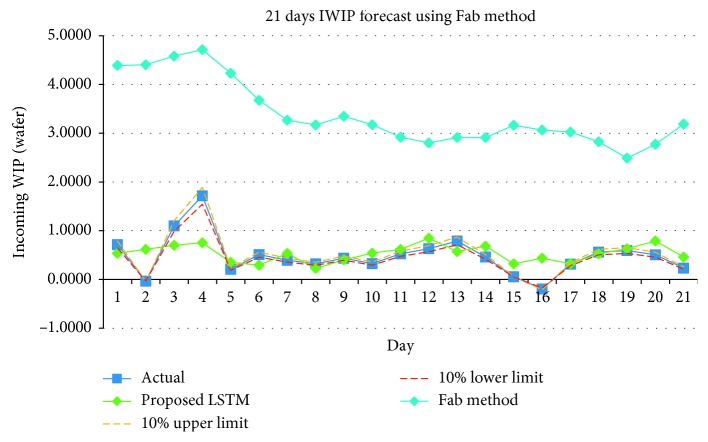
IWIP forecast using Fab method.

We then compare the forecast result using Combination 3 to the statistical forecasting method used in the Fab. In order to make the writing clearer, the statistical forecasting method used in the Fab is abbreviated as Fab method. Tables 13 and 14 tabulate the hit rate and Pearson's *r* of Fab's method, respectively.  Table 15 tabulates the comparison of the forecast results between the proposed method and Fab's method.  Figure 16 shows the WIP forecast using Fab's method.

The Fab method serves as the baseline to measure the performance of the LSTM forecasting model. From the results tabulated in Table 15, the proposed method with LSTM forecasting model outperformed the Fab method. However, both hit rate and Pearson's *r* of the proposed method is unable to remain consistent for three consecutive weeks forecasted. The results also show that the IWIP forecasted by the Fab method consistently failed to meet the requirement of the Fab for both hit rate and Pearson's *r*. The main reason for the inaccuracy of the Fab method is that it only considers for products with the number of wafers ordered dominating the total WIP in the production line. However, operators need to process other wafers from other products as well. Hence, the wafers did not arrive on time as predicted. In addition, the Fab method does not consider the number of tools available at each process steps to process the wafers, and the total amount of time that each tool is used to process the wafers. In real environment, a tool can be taken offline for maintenance purposes or it could be used by the respective engineers to process specially crafted wafers for research and development purposes. Without taking into these considerations, the Fab method indirectly assumed that the number of tools available, and the time of each tool dedicated to process wafers are the same across the entire period of the wafer fabrication process. This assumption caused the forecasted results to have negative correlation with the actual IWIP.

For hit rate, the proposed method only scored 50% for week 3 while for Pearson's *r*, the proposed method only scored 0.31 for week 2. One of the factors that caused the reduced performance of the model could be due to the reason that the size of the historical data to train the LSTM model is not large enough. Larger historical IWIP data could potentially allow the LSTM model of the proposed method to discover more time-dependent relations in the Fab's production environment. With the additional time-dependent relations discovered, the accuracy of the model's forecasting can be increased.

The next factor that could contribute to the inconsistent result of the model is the limited number of features that used to represent Fab's production environment. Having additional features to represent Fab's production environment could allow LSTM to perform better modeling of WIP arrival. The examples of additional data that could serve as such features are the actual number of equipment that supplies the WIP to the tool group of interest, the amount of time each equipment in the tool group is processing the production wafers instead of performing other maintenance activities, and the number of wafers that each equipment in the tool group of interest has actually processed.

The last factor that contributes to the inconsistent results could be the need for more hidden layers. As the number of hidden layers increases, it creates a deeper neural network that could potential allow the model to capture even more time-dependent relations in the data. However, in order to benefit from deeper neural network, larger dataset must first be obtained so that the model can be properly trained.

For the experiments conducted, the selection of sizes for the LSTM model's parameters and the number of experimental runs is largely affected by the hardware resources allocation and the software capability setup. From the hardware resource perspective, sufficient CPU should be allocated in the computing machine, while from the software capability perspective, parallelization should be enabled to fully utilize the available CPU. With 4 CPUs allocated in a virtual machine environment and parallelization enabled in Keras, it took approximately 8 hours to complete one full experiment. One full experiment refers to the complete evaluation all the predefined sizes. For real production deployment, 8 hours is too long to obtain a usable model. Parallelization with sufficient number of CPUs in the computing machine are therefore critical in the production environment as the results should be obtained as fast as possible in order for the managements to make the necessary decision for production line stability. Hence, proper hardware planning is required for production deployment.

## 5. Conclusion

PM activity is an important activity in the Fab as it maintains or increases the operational efficiency and reliability of the tool. Proper PM planning is necessary as PM activity takes significantly long time to complete, thus it is desirable to perform this activity when the IWIP to the tool group is expected to be low. With an IWIP prediction model that is capable to predict the IWIP with high accuracy, PM activity can be planned and managed better to reduce its negative impact to the fab's CT. Reducing the negative impact to CT is important as this will enable the Fab to meet the On-Time-Delivery (OTD) committed to customers. With consistency in the OTD, the logistic management of the company can be improved as well, such as proper storage place planning to keep the fabricated wafers and scheduling their transportations for shipments. Well-planned PM activities also allow better manpower planning in areas such as manpower planning. When performing the PM activity, sufficient tool engineers and tool vendors are required to be onsite to perform the prescribed maintenance activities. Well-planned PM activities allow the required manpower to be properly prepared. Well-planned manpower directly contributes to better manpower cost planning. With proper PM planning in-place, tools in the Fab can be scheduled to receive their proper maintenances on time. It is important for tools in the Fab to receive their appropriate maintenances on-time to improve its productivity and lifetime extension. With improved performance and extended lifetime, the capital investments of the company on the tools can be optimized. Reliable tool performance will also increase the trust of the customers as chances of the fabricated wafers being scrapped due to unhealthy tool are minimized.

In this paper, we investigated LSTM to assist in PM planning in Fab by predicting the IWIP to a tool group. The comparison of the performance of the proposed method was done with an existing forecasting method from the Fab. The proposed method was trained using the historical IWIP data provided by the Fab, which is a time series data. Both hit rate and Pearson's correlation coefficient are important criteria that determine the forecast capability. The proposed method has demonstrated results that outperformed the Fab method by reaching above the requirement of the Fab for week 1 and week 3, while the Fab method fails to meet Fab's requirement for all three weeks. In terms of hit rate, the proposed method shows higher percentage than Fab's method. Following the requirement given by the Fab, the results of the proposed method signifies that for forecast duration of seven days, it is able to identify more accurately the two days that the IWIP will be highest and the two days the IWIP will be lowest in a week. In terms of Pearson's correlation coefficient, *r*, the proposed method shows positive correlation and higher value than the Fab's method. This result signifies that the forecast results of the proposed method are able to produce better forecasting results that have closer proportional changes in relation to the actual IWIP. The LSTM model used in the proposed method contains memory cells to memorize the long and short temporal features in the data, which yields better performance for the prediction of time series data. Therefore, the proposed method will be very useful and benefit to the PM planning.

Although the proposed method is outperformed the existing Fab's statistical method, there is still room for improvement. The first future work is to increase the size of historical dataset. With the use of larger historical dataset that spans across longer historical time horizon to train the LSTM model, it may be potential for the LSTM model to discover significant WIP arrival pattern that could have been missed out in smaller historical dataset. The second future work is to extend the univariate forecasting model in this research to multivariate forecasting model. The reason for this extension is to allow the inclusion of more features to train the LSTM model so that the LSTM model can better model the actual environment of the Fab. The next future work is to increase the number of hidden layers in the LSTM forecasting model. The approach to increase the number of hidden layer is also an initial step to experiment the potential use of deep-learning model in time series forecasting. The last future work is to extend the application of the proposed method to predict the IWIP of other types of tool group to experiment if the proposed method is capable to delivering the same prediction performance. The collected prediction results across various types of tool groups from the future work will also allow us to generalize the proposed method to be used as a generic IWIP prediction model for the fab.

## Figures and Tables

**Table 1:** Existing statistical WIP forecasting steps.

Step	Description
1	Given the process flow name of product *P* is *F*, retrieve all process steps for *F*
2	For each process step *s* in *F*, get the turn-around-time of *s*, TAT_*s*_; this value is predefined in the manufacturing execution system (MES) of the fab.
3	Let *L*_F_ denotes the total photolithography layers in *F*, *l*_F_ denotes the number of photolithography layers in *F* completed per day, the day-per-mask-layer (DPML) committed for *P*, DPML_P_ is denoted as DPML_P_=(1/*l*_F_) *TAT*_*F*_ denotes the total turn-around-time (TAT) for *F*, denoted as TAT_F_=∑_*s*=1_^*F*^TAT_*s*_ The run rate for *F*, RR_F_ is RR_F_=((L_F_ × DPML_P_)/TAT_F_)
4	Cycle time (CT) for a process step *s*, CT_*s*_ is CT_*s*_= RR_F_ × TAT_*s*_
5	For each lot, sum the next *n* steps of *s* until the CT reaches 24 hours. The last *s* would be the forecasted destination step of the lot after 24 hours
6	To forecast the destination step of the lot for the next *D* day, sum the next *n* steps of *s* until the CT reaches *D* × 24 hours. The last *s* would be the forecasted destination step of the lot for the next *D* day

**Table 2:** Data formation for training dataset.

Set	Training dataset		
	*X*	*Y*	*Z*
1	(*x*_1_, *x*_2_, *x*_3_, *x*_4_, *x*_5_, *x*_6_, *x*_7_)	7	1
2	(*x*_2_, *x*_3_, *x*_4_, *x*_5_, *x*_6_, *x*_7_, *x*_8_)	7	1
3	(*x*_3_, *x*_4_, *x*_5_, *x*_6_, *x*_7_, *x*_8_, *x*_9_)	7	1
4	(*x*_4_, *x*_5_, *x*_6_, *x*_7_, *x*_8_, *x*_9_, *x*_10_)	7	1
5	(*x*_5_, *x*_6_, *x*_7_, *x*_8_, *x*_9_, *x*_10_, *x*_11_)	7	1
6	(*x*_6_, *x*_7_, *x*_8_, *x*_9_, *x*_10_, *x*_11_, *x*_12_)	7	1
7	(*x*_7_, *x*_8_, *x*_9_, *x*_10_, *x*_11_, *x*_12_, *x*_13_)	7	1

**Table 3:** Data formation for testing dataset.

Set	Testing dataset	
	*X*	*Y*
1	(*x*_8_, *x*_9_, *x*_10_, *x*_11_, *x*_12_, *x*_13_, *x*_14_)	1
2	(*x*_9_, *x*_10_, *x*_11_, *x*_12_, *x*_13_, *x*_14_, *x*_15_)	1
3	(*x*_10_, *x*_11_, *x*_12_, *x*_13_, *x*_14_, *x*_15_, *x*_16_)	1
4	(*x*_11_, *x*_12_, *x*_13_, *x*_14_, *x*_15_, *x*_16_, *x*_17_)	1
5	(*x*_12_, *x*_13_, *x*_14_, *x*_15_, *x*_16_, *x*_17_, *x*_18_)	1
6	(*x*_13_, *x*_14_, *x*_15_, *x*_16_, *x*_17_, *x*_18_, *x*_19_)	1
7	(*x*_14_, *x*_15_, *x*_16_, *x*_17_, *x*_18_, *x*_19_, *x*_20_)	1

**Table 4:** Parameter size selection results.

Combination	Parameter				RMSE
	Epoch	Batch size	*n* LSTM hidden layers stacked	LSTM neuronsize	
1	100	10	3	512, 8, 8	0.0096
2	100	10	3	512, 8, 16	0.0086
3	100	20	3	512, 16, 16	0.0091

**Table 5:** Hit rate for Combination 1.

Week	*f*	Actual	Forecast	Hit
w1	1			
	2	L		
	3	H	H	1
	4	H	H	1
	5	L	L	1
	6		L	
	7			
				HR = 75%

w2	1		L	
	2	L		
	3			
	4	L	H	
	5	H	L	
	6			
	7	H	H	1
				HR = 25%

w3	1	L	L	1
	2	L		
	3		L	
	4	H		
	5	H	H	1
	6		H	
	7			
				HR = 50%

**Table 6:** Hit rate for Combination 2.

Week	*f*	Actual	Forecast	Hit
w1	1			
	2	L		
	3	H	H	1
	4	H	H	1
	5	L	L	1
	6		L	
	7			
				HR = 75%

w2	1		L	
	2	L	L	1
	3			
	4	L		
	5	H	H	1
	6		H	
	7	H		
				HR = 50%

w3	1	L		
	2	L		
	3		L	
	4	H		
	5	H	H	1
	6		H	
	7		L	
				HR = 25%

**Table 7:** Hit rate for Combination 3.

Week	*f*	Actual	Forecast	Hit
w1	1			
	2	L		
	3	H	H	1
	4	H	H	1
	5	L	L	1
	6		L	
	7			
				HR = 75%

w2	1			
	2	L	L	1
	3			
	4	L		
	5	H	H	1
	6			
	7	H	H	1
				HR = 75%

w3	1	L	L	1
	2	L		
	3		L	
	4	H		
	5	H	H	1
	6		H	
	7			
				HR = 50%

**Table 8:** Pearson's *r* for combination 1.

Week	*r*
w1	0.31
w2	0.06
w3	0.34

**Table 9:** Pearson's *r* for combination 2.

Week	*r*
w1	0.40
w2	0.28
w3	0.46

**Table 10:** Pearson's *r* for combination 3.

Week	*r*
w1	0.42
w2	0.31
w3	0.43

**Table 11:** Summary of hit rate for Combinations 1, 2, and 3.

Combination	Hit rate (%)		
	w1	w2	w3
1	75	25	50
2	75	50	25
3	75	75	50

**Table 12:** Summary of Pearson's *r* for combinations 1, 2, and 3.

Combination	Pearson's *r*		
	w1	w2	w3
1	0.31	0.06	0.34
2	0.40	0.28	0.46
3	0.42	0.31	0.43

**Table 13:** Hit rate for Fab method.

Week	*f*	Actual	Forecast	Hit
w1	1			
	2	L		
	3	H	H	1
	4	H	H	1
	5	L		
	6		L	
	7		L	
				HR = 50%

w2	1			
	2	L	H	
	3		H	
	4	L		
	5	H	L	
	6		L	
	7	H		
				HR = 0%

w3	1	L	H	
	2	L		
	3			
	4	H		
	5	H	L	
	6		L	
	7		H	
				HR = 0%

**Table 14:** Pearson's *r* for Fab method.

Week	*r*
w1	0.28
w2	−0.11
w3	−0.82

**Table 15:** Forecast result comparison between proposed method and Fab.

	Hit rate (%)			Pearson's *r*		
	Week			Week		
	w1	w2	w3	w1	w2	w3
Proposed method	75.0	75.0	50.0	0.42	0.31	0.43
Fab	50.0	0.0	0.0	0.28	−0.11	−0.82

## Data Availability

The time-series data used to support the findings of this study were supplied by X-Fab Sarawak Sdn. Bhd. under privacy agreement, and there, the data cannot be made freely available. The data potentially reveal sensitive information, and therefore, their access is being restricted.
